# Overexpression of thioredoxin m in chloroplasts alters carbon and nitrogen partitioning in tobacco

**DOI:** 10.1093/jxb/erab193

**Published:** 2021-05-08

**Authors:** María Ancín, Luis Larraya, Igor Florez-Sarasa, Camille Bénard, Alicia Fernández-San Millán, Jon Veramendi, Yves Gibon, Alisdair R Fernie, Iker Aranjuelo, Inmaculada Farran

**Affiliations:** 1 Institute for Multidisciplinary Applied Biology (IMAB), Dpto. Agronomía, Biotecnología y Alimentación, Universidad Publica de Navarra (UPNA), Campus Arrosadia, 31006 Pamplona, Spain; 2 Max-Planck-Institut für Molekulare Pflanzenphysiologie, Am Mühlenberg 1, 14476 Potsdam-Golm, Germany; 3 Centre for Research in Agricultural Genomics (CRAG) CSIC-IRTA-UAB-UB, Campus UAB Bellaterra, 08193 Barcelona, Spain; 4 UMR 1332 Biologie du Fruit et Pathologie and Plateforme Metabolome Bordeaux, INRA, Bordeaux University, 33882 Villenave d’Ornon, France; 5 Instituto de Agrobiotecnología, CSIC-Gobierno de Navarra, Avda. Pamplona 123, 31192 Mutilva, Spain; 6 Pontificia Universidad Católica de Chile, Chile

**Keywords:** Carbon metabolism, chloroplast, glutamine synthetase, GS-GOGAT pathway, nitrogen metabolism, photorespiration, thioredoxin

## Abstract

In plants, there is a complex interaction between carbon (C) and nitrogen (N) metabolism, and its coordination is fundamental for plant growth and development. Here, we studied the influence of thioredoxin (Trx) m on C and N partitioning using tobacco plants overexpressing Trx m from the chloroplast genome. The transgenic plants showed altered metabolism of C (lower leaf starch and soluble sugar accumulation) and N (with higher amounts of amino acids and soluble protein), which pointed to an activation of N metabolism at the expense of carbohydrates. To further delineate the effect of Trx m overexpression, metabolomic and enzymatic analyses were performed on these plants. These results showed an up-regulation of the glutamine synthetase–glutamate synthase pathway; specifically tobacco plants overexpressing Trx m displayed increased activity and stability of glutamine synthetase. Moreover, higher photorespiration and nitrate accumulation were observed in these plants relative to untransformed control plants, indicating that overexpression of Trx m favors the photorespiratory N cycle rather than primary nitrate assimilation. Taken together, our results reveal the importance of Trx m as a molecular mediator of N metabolism in plant chloroplasts.

## Teaser

Thioredoxin m overexpression modulates carbon and nitrogen partitioning in plants by regulating the photorespiratory nitrogen cycle, where an increase of activity and stability of glutamine synthetase was shown.

## Introduction

The carbon (C) and nitrogen (N) metabolisms of higher plants are tightly coordinated to maintain optimal growth and development. This strong connection ensures that amino acids and C skeletons are available for plant metabolic requirements, which entails that photosynthetic products must be partitioned between carbohydrate and amino acid biosynthesis. This partitioning is controlled by an extremely complex network involving signals from various metabolites, proteins, and hormones. Moreover, the redox status of the cell has been proposed as an important factor that might impact the integration of plant C and N assimilation (Nunes-Nesi *et al.*, 2010).

Within chloroplasts, two thiol-based redox regulatory systems function in parallel: (i) the ferredoxin-thioredoxin (Fd-Trx) system, which depends on photosynthetically reduced Fd to supply electrons to the Fd-Trx reductase, which in turn reduces Trxs; and (ii) the NADPH-dependent Trx reductase type C (NTRC), which contains both Trx-reductase and Trx domains in a single polypeptide, and is reduced by NADPH. Plastid classical Trxs transfer reducing power to target enzymes through the action of Fd-Trx reductase, modulating their activity related to various metabolic pathways. Five Trx types, termed f, m, x, y, and z, have been found within the chloroplast. Originally, f- and m-type Trxs were identified as regulators of thiol enzymes involved in photosynthetic C assimilation in the chloroplast (Wolosiuk and Buchanan, 1977; Buchanan, 1980), whereas x-, y-, and z-type Trxs have been more closely linked to the regulation of antioxidant enzymes (Meyer *et al.*, 2012). However, proteomic approaches revealed a great number of chloroplast-localized Trx targets that basically cover all the main chloroplast functions (Montrichard *et al.*, 2009). More recently, genetic studies have been used to dissect specific roles for plastid Trxs *in vivo* (Geigenberger and Fernie, 2014; Geigenberger *et al.*, 2017; Nikkanen *et al.*, 2017). According to previous studies, Trx f is mainly associated with the redox regulation of C reactions, including enzymes of both the Calvin–Benson cycle (CBC) and starch metabolism (Collin *et al.*, 2003; Marri *et al.*, 2009; Sanz-Barrio *et al.*, 2013; Thormählen *et al.*, 2013; Yoshida *et al.*, 2015; Naranjo *et al.*, 2016). By contrast, Trx m is proposed to control a wider range of processes, such as the biogenesis of Cyt*b*_*6*_*f* and PSII (Motohashi and Hisabori, 2010; Wang *et al.*, 2013), cyclic electron flow around PSI (Courteille *et al.*, 2013), tetrapyrrole synthesis (Luo *et al.*, 2012), export of reducing power via the malate valve (Thormählen *et al.*, 2017), non-photochemical quenching-dependent photoprotection (Da *et al*., 2018), and the activation of antioxidant (Collin *et al.*, 2003; Rey *et al.*, 2013) and CBC (Okegawa and Motohashi, 2015) enzymes. However, the involvement of plastid Trxs in the regulation of N metabolism and, more precisely, N assimilation and partitioning has been poorly studied.

Previous studies have suggested a role of Trxs in the regulation of N assimilation via redox regulation of chloroplast glutamine synthetase (GS2) and Fd-dependent glutamate synthase (Fd-GOGAT) in algae (Schmidt, 1981; Tischner and Schmidt, 1982; Florencio *et al.*, 1993) and cyanobacteria (Papen and Bothe, 1984; Lindahl and Florencio, 2003). Fd-GOGAT catalyzes the conversion of glutamine (Gln) and 2-oxoglutarate to two molecules of glutamate (Glu), whereas GS2 catalyzes the ATP-dependent formation of Gln from Glu and ammonia, thus participating in primary N assimilation as well as in the reassimilation of ammonia released from a variety of metabolic pathways such as photorespiration, catabolism of amino acids, and metabolism of phenylpropanoids (Foyer *et al.*, 2006). The two enzymes constitute the GS-GOGAT cycle, which is essential for N assimilation and photorespiration in chloroplasts. *In vitro* experiments have also provided evidence that these two enzymes may be subjected to redox regulation in higher plants: (i) spinach and soybean Fd-GOGAT enzymes were reported to be preferentially activated by Trx m (Lichter and Häberlein, 1998); and (ii) the activity of GS2 has been suggested to be directly redox modulated in chloroplasts (Choi *et al.*, 1999). Moreover, both enzymes were identified as Trx targets in different proteomic studies (Montrichard *et al.*, 2009), and a redox regulation of the GS-GOGAT cycle *in vivo* was suggested to take place in Arabidopsis leaves treated with the reductant DTT (Kolbe *et al.*,2006).

The present study aims to shed light on the Trx-mediated redox regulation of N metabolism in plant leaves. Previous work has indicated that the altered redox status of tobacco chloroplasts achieved by the overexpression of Trx m might impair the cell C status (Sanz-Barrio *et al.*, 2013), involving processes such as photosynthesis (Courteille *et al.*, 2013) and chloroplast redox homeostasis (Rey *et al.*, 2013). However, the effect of Trx m overexpression on C and N metabolism still remains to be analyzed. Here, we conducted a wide metabolomic characterization of Trx m-overexpressing tobacco plants, concluding that Trx m alters C and N partitioning by promoting N instead of C metabolism. Moreover, a putative role of Trx m in GS2 redox protection, linked to its effect on the photorespiratory N cycle, is discussed. Taken together, our results suggest a role for Trx m in controlling N metabolism in plant chloroplasts.

## Materials and methods

### Plant material and growth conditions

The study was carried out with tobacco (*Nicotiana tabacum* cv. Petit Havana SR1) wild-type (Wt) plants and transplastomic plants overexpressing the mature *Trxm* coding sequence (hereafter termed the o/exTrxm genotype) from the chloroplast genome under the control of the *rrnG10*L promoter (Sanz-Barrio *et al.*, 2013). Because insertion of transgenes into plastid DNA occurs via homologous recombination at a specific insertion site, all transplastomic lines are identical (Bock, 2015) and therefore a single transplastomic line was used. Two-week-old seedlings, germinated *in vitro*, were transferred to 3 litre pots filled with soil and grown in a phytotron under a 16 h light/8 h dark photoperiod with 150 µmol m^–2^ s^–1^ photosynthetic photon flux density, at 28 °C. Plants were watered twice a week with 0.5× Hoagland nutrient solution. To test the behavior of o/exTrxm plants under N-limiting conditions, a second experiment was conducted. In this case, plants were grown under hydroponic conditions in 3 litre pots filled with an inert substrate such as perlite. Plants were irrigated daily with nutrient solution [3 mM KH_2_PO_4_, 1 mM MgSO_4_, 1 mM CaCl_2_ and 2 g l^–1^ Nutrel C commercial micronutrient mixture (Yara Iberian, Madrid, Spain), pH 5.8–6.2] containing 1 mM or 2 mM KNO_3_ (Geiger *et al.*, 1999). For the 1 mM KNO_3_ treatment, 1 mM KCl was added to supplement potassium in line with the 2 mM KNO_3_ treatment. Pots were washed every week with distilled water to prevent salt accumulation. All analyses were carried out on the youngest fully expanded leaves of 6-week-old plants. Whole plants, including leaves, stems, and roots, were harvested and dried in an oven at 60 °C for 72 hours, and total biomass [g dry weight (DW)] was determined.

### Trx m immunoblot analysis

For quantification of Trx m, proteins from leaf samples were extracted and blotted using specific anti-Trx m antibody (Sanz-Barrio *et al.*, 2013). Total protein was extracted with extraction buffer (125 mM Tris–HCl, pH 6.8, 4% SDS, and 20% glycerol), and quantified using the RC-DC protein assay (Bio-Rad, Hercules, CA, USA) with bovine serum albumin as a standard, according to the manufacturer’s instructions. Overexpressed Trx m in leaf extracts was quantified by western blotting using serial dilution of Trx m protein produced in *Escherichia coli* as a standard. Immunoblots were quantified using the GeneTools analyzer software (SynGene, Cambridge, UK).

Determination of the Trx m redox state *in vivo* was performed in leaves of plants overexpressing Trx m in light and dark conditions. Leaves were ground in liquid N_2_, and protein was extracted and incubated with or without 50 mM DTT (reducing agent) or 50mM diamide or H_2_O_2_ (oxidant agents). Extracts were precipitated with 10% (w/v) trichloroacetic acid and washed twice with 100% ice-cold acetone before the protein pellet was resuspended in 2% SDS and 150 mM Tris–HCl, pH 7.5, buffer. One portion of the samples was incubated with 10 mM 4-acetamido-4′-maleimidylstilbene-2,2′-disulfonate (AMS) (Invitrogen, ThermoFisher Scientific) for 1 h at room temperature in the dark, to alkylate cysteine (Cys) residues. Both AMS-treated and untreated samples were separated by 15% non-reducing SDS-PAGE and immunoblotted with anti-Trx m specific antibody (1:5000). The *in vivo* reduced form becomes labelled with AMS and migrates more slowly in SDS-PAGE.

### Determination of starch, soluble sugars, protein, and amino acids

The concentrations of soluble sugars and starch were determined after 4 h light and at the end of the light and dark periods in tobacco leaf ethanol extracts and pellets by using, respectively, HPLC and an amyloglucosidase-based test kit (R-Biopharm AG, Darmstadt, Germany) as previously described (Sanz-Barrio *et al.*, 2013). For determination of protein concentrations, leaf samples (100 mg) harvested after 4 h illumination were ground in liquid N_2_, resuspended in 200 μl of soluble protein extraction buffer (100 mM Tricine–NaOH, pH 8, 8 mM MgCl_2_, and 2 mM EDTA) and incubated for 15 min on ice. The supernatant was obtained after 5 min of centrifugation at 10 000 *g* at 4 °C and the concentration of soluble protein was measured with an RC-DC protein assay (Bio-Rad) using bovine serum albumin as a standard. For amino acid quantification, leaf samples harvested after 4 h illumination (50 mg) were extracted with ethanol, derivatized, and analyzed on a reverse-phase HPLC column using the fluorescing reagent 6-aminoquinolyl-*N*-hydroxysuccinimidyl carbamate (AQC) (Loiret *et al.*, 2009).

### Metabolomics

Frozen leaf samples harvested after 4 h illumination were ground in 2 ml microcentrifuge tubes using stainless steel beads and a Retsch Mixer Mill MM 400 (Retsch, Haan, Germany) under cryogenic conditions. From these samples, metabolites were extracted, derivatized, and analyzed via gas chromatography–time-of-flight mass spectrometry (GC-TOF-MS) analyses as described before (Lisec *et al.*, 2006). The GC-TOF-MS system consisted of a CTC CombiPAL autosampler (CTC Analytics, Zwingen, Switzerland), an Agilent 6890N gas chromatograph (Agilent Technologies, Santa Clara, CA, USA) and a LECO Pegasus III time-of-flight mass spectrometer running in EI+ mode (Leco Instruments, St. Joseph, MI, USA). Metabolites were identified by comparison with database entries of authentic standards (Kopka *et al.*, 2005; Schauer *et al.*, 2005) using TagFinder software (Luedemann *et al.*, 2012). The peak intensity of a representative fragment was normalized with that of the internal standard ribitol and sample fresh weight (FW) and referred to as relative abundance. The parameters used for the peak annotation are listed in Supplementary Table S1 according to Fernie *et al.* (2011).

### Enzyme assays

Leaves harvested after 4 h illumination were immediately frozen and ground in liquid N_2_. Aliquots of ~20 mg FW were extracted with 500 µl of extraction buffer according to Gibon *et al.* (2004). Phosphoglycerokinase (PGK), NADP-dependent glyceraldehyde-3-phosphate dehydrogenase (NADP-GAPDH), fructose-bisphosphatase (FBPase), GS, Fd-GOGAT, NAD-dependent glutamate dehydrogenase (NAD-GDH), and phospho*enol*pyruvate carboxylase (PEPC) were assayed following Gibon *et al.* (2004), and triose-phosphate isomerase (TPI) was assayed according to Biais *et al.* (2014). All assays were performed on microplates using 96-head pipetting robots (Hamilton, Villebon-sur-Yvette, France) and UV-visible readers (SAFAS, Monaco, France). Assay optimization and calculations were conducted as described in Bénard and Gibon (2016).

### RNA extraction and RT–qPCR experiments

A 1 µg sample of DNAse-treated total RNA, extracted from tobacco leaves with Trizol® reagent (Invitrogen), was used for cDNA synthesis by reverse transcription (RT) with a PrimeScript™ RT–PCR Kit (Takara Bio, Kusatsu, Japan) according to the manufacturer’s instructions. Quantitative PCR (qPCR) analysis was carried out using gene-specific primers listed in Supplementary Fig. S1, and was conducted in three biological replicates. The analysis was performed with SYBR® Premix Ex Taq™ (Takara Bio), and the comparative threshold cycles (Ct) method (ΔΔCt) was applied for relative quantification of gene expression using an AriaMx real-time PCR System (Agilent Technologies). Relative expression levels were normalized against *16s rRNA* and *actin* as reference genes.

### Chlorophyll, nitrate, and ammonium quantification

Photosynthetic pigments were extracted from leaf disks collected from fully expanded leaves and crushed in 5 ml of 80% acetone. After centrifugation at 10 000 *g* for 5 min at 4 °C, the amount of Chl *a* and *b* was measured spectrophotometrically and calculated according to Lichtenthaler (1987).

Aliquots of ~30 mg of frozen leaf material were homogenized with 600 μl of ultrapure water. Samples were then incubated at 80 °C for 5 min and centrifuged at 20 000 *g* and 4 °C for 20 min. The supernatant was recovered and used for nitrate (Cataldo *et al.*, 1975) and ammonium (Sarasketa *et al.*, 2014) determination.

### Gas exchange and chlorophyll fluorescence determinations

Gas exchange measurements were carried out on fully expanded leaves with the LI6400XT gas exchange portable photosynthesis system (LI-COR, Lincoln, NE, USA). Determinations were conducted between 4 h and 7 h after the onset of illumination. The light-saturated rate of CO_2_ assimilation was measured at 1000 µmol m^–2^ s^–1^ photosynthetic photon flux density, with 400 μmol s^−1^ air flow rate, a temperature of 25 °C, and 60% relative humidity. Photosynthetic parameters were obtained using the equations of von Caemmerer and Farquhar (1981). After performing the net photosynthesis determinations under ambient CO_2_ and O_2_ conditions, leaves were kept in the chamber and N_2_ from a tank (Air Liquide, Paris, France) was plugged into the inlet of the Li-6400XT to remove O_2_ from the air entering the leaf chamber, to allow photorespiration determinations (A_N2%_). Net photosynthesis at high O_2_ concentration (A_N40%_) was measured when a tank containing 40% O_2_ was plugged into the inlet of the Li-6400XT. Photorespiration was expressed as the percentage reduction in the net photosynthetic rate by photorespiration using the equation: (A_N2%_–A_N21%_)/A_N2%_×100 (Florez-Sarasa *et al.*, 2019). The measurements of dark respiration (R_d_) were performed at least 20 min after the lights were switched off to avoid light-enhanced dark respiration, and thus to obtain a proper R_d_ measurement.

### Redox-dependent structure and activity of glutamine synthetase

Protein complexes were analyzed from intact chloroplasts solubilized with 1.5% digitonin by Blue Native (BN)-PAGE using a 4–16% Native PAGE Novex Bis-Tris gel (Invitrogen) according to the manufacturer’s instructions. After electrophoresis, protein complexes were transferred to a polyvinylidene fluoride (PVDF) membrane and probed with anti-GS2 antibody (AS08 296, Agrisera, Vännäs, Sweden). Protein samples were incubated for 30 min on ice in the presence or absence of 10 mM DTT or 1 mM CuCl_2_ before loading.

Determination of GS redox-dependent activity was performed, as previously described (Scheible *et al.*, 1997), on protein extracts from Wt plants incubated with or without 5 mM DTT or 100 µM CuCl_2_ for 30 min on ice. Assessment of the recovery of GS activity by reduction was performed by incubating oxidized samples (after treatment with 100 µM CuCl_2_) with 5 mM DTT for 30 min on ice.

### Quantification and stability of GS2 protein

For quantification of GS2, ground leaf samples were homogenized in 2 volumes of total protein extraction buffer (125 mM Tris–HCl, pH 6.8, 4% SDS, and 20% glycerol). Samples were incubated on ice for 15 min and then centrifuged at 10 000 *g* for 5 min at 4 °C, and the supernatant was recovered. Protein extracts were quantified by a RC-DC protein assay (Bio-Rad), and 2.5 µg samples were separated by SDS–PAGE on 10% polyacrylamide gels and transferred to a PVDF membrane for immunoblotting with anti-GS2 antibody. Immunoblots were quantified using the GeneTools analyzer software (SynGene).

Determination of the stability of GS2 protein was performed essentially as described in Ortega *et al.* (1999) with some modifications. Leaves of Wt and o/exTrxm plants were ground in liquid N_2_ and homogenized with three volumes of extraction buffer [50 mM Tris–HCl, pH 8.0, 20% (v/v) glycerol, 1 mM MgCl_2_, 1 mM DTT, and protease inhibitor cocktail]. The homogenate was centrifuged at 20 000 *g* for 15 min at 4 °C. The supernatant was incubated in a reaction containing 50 mM imidazole, pH 7.4, 0.125 mM FeCl_3_ and 5 mM DTT, for 24 h at 30 °C. Aliquots were taken at several times and analyzed by western blot. The experiment was repeated with eight biological replicates, and a representative immunoblot is shown.

## Results

### Overexpression of Trx m alters the balance of C and N in tobacco plants

**Fig. 1.**
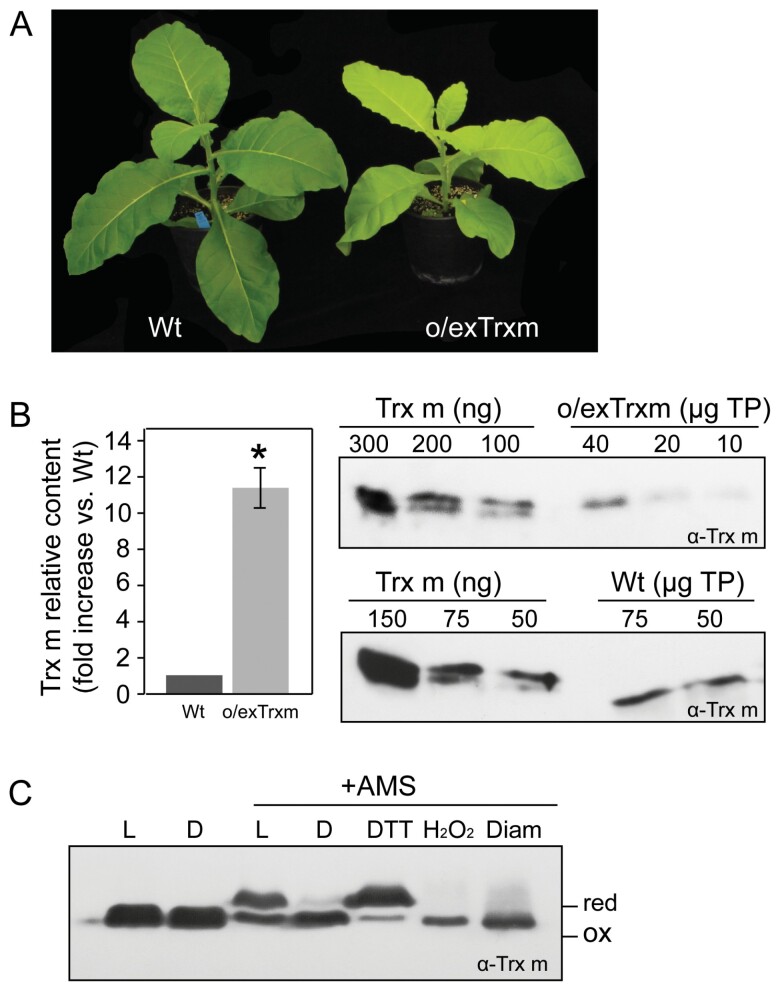
Trx m overexpression and redox state in tobacco plants. (A) Phenotype of transplastomic o/exTrxm and Wt plants. (B) Trx m accumulation in transplastomic tobacco plants. Quantitation of Trx m protein was done by densitometric analysis of a dilution series of recombinant Trx m from *E. coli* and leaf extracts from o/exTrxm and Wt plants. Representative western blot analyses of Trx m expression are shown. The amount of plant extract loaded, indicated over the blots, was adjusted to be comparable to the signal of the recombinant Trx m used as a standard TP, total protein. Results are presented as the fold increase of Trx m content in o/exTrxm genotype relative to the Wt (=1). Results are the mean ±SE of three independent plants per genotype. Statistical significance compared with the Wt is indicated by an asterisk (*P<0.05, Student’s *t*-test). (C) Redox status of overexpressed Trx m in tobacco chloroplasts. AMS alkylation and western blot analysis after non-reducing SDS-PAGE of Trx m (specific antibody was used). Protein leaf extracts incubated with DTT, H_2_O_2_, or diamide (Diam) before treatment with AMS were used as reduced and oxidized controls. Protein extracts not incubated with AMS were used as non-alkylated controls. The mobility of the alkylated (reduced; red) and non-alkylated (oxidized; ox) forms is indicated.

We first characterized the tobacco plants overexpressing the endogenous *Trxm* gene from the plastid genome (o/exTrxm plants) grown in a phytotron under a 16 h light/8 h dark regime with 150 µmol m^–2^ s^–1^ photosynthetic photon flux density, at 28 °C. Plants of this genotype showed slower growth than the Wt and pale-green colored leaves (Fig. 1A), a phenotype similar to that previously reported (Rey *et al.*, 2013; Sanz-Barrio *et al.*, 2013; Ancín *et al.*, 2019). In the described growing conditions, o/exTrxm plants accumulated Trx m protein at levels around 12-fold higher than that of Wt plants (Fig. 1B). A significant portion of the overexpressed protein was reduced in the o/exTrxm genotype in the light (Fig. 1C), and presumably was active and able to transfer reducing equivalents to its target enzymes.

**Fig. 2.**
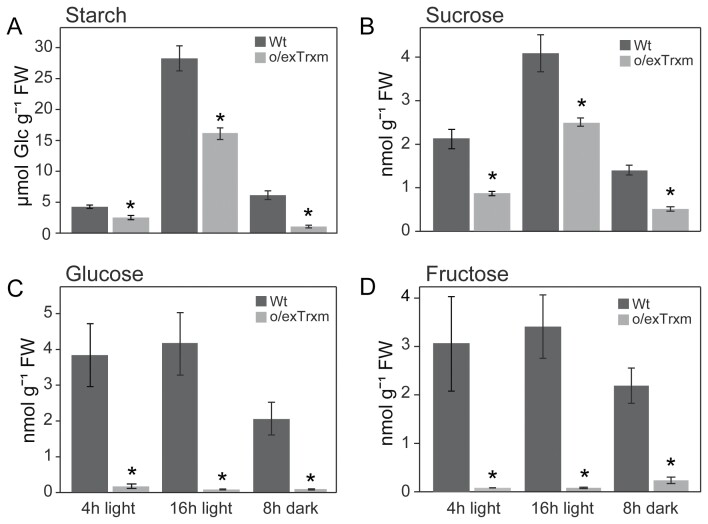
Starch and sugar concentrations in Wt and o/exTrxm tobacco leaves. (A) Starch, (B) sucrose, (C) glucose, and (D) fructose accumulation in young fully expanded leaves of phytotron-grown plants harvested after 4 h light, 16 h light, and 8 h dark periods. Results are the mean ±SE of six individual plants. Statistical significance compared with Wt plants is indicated by asterisks (*P<0.05, Student’s *t*-test).

We then investigated the accumulation of C and N-derived metabolites in the o/exTrxm genotype. Concerning C-derived metabolites, starch concentrations were significantly decreased in o/exTrxm leaves relative to the Wt, by ~40% after both 4 h and 16 h of light, while an 80% decrease was observed at the end of the 8 h dark period (Fig. 2A). Likewise, the sucrose concentration was significantly lower in the transplastomic line, showing a quite similar pattern to that of starch (Fig. 2B). Furthermore, o/exTrxm leaves showed a dramatic decrease in the glucose and fructose concentrations compared with the Wt, with the concentrations of these metabolites being very similar in the light and dark (Fig. 2C, D). These results indicate that, as suspected from previous works (Sanz-Barrio *et al.*, 2013), the leaf carbohydrate metabolism was impaired by the overexpression of Trx m in tobacco chloroplasts.

**Fig. 3.**
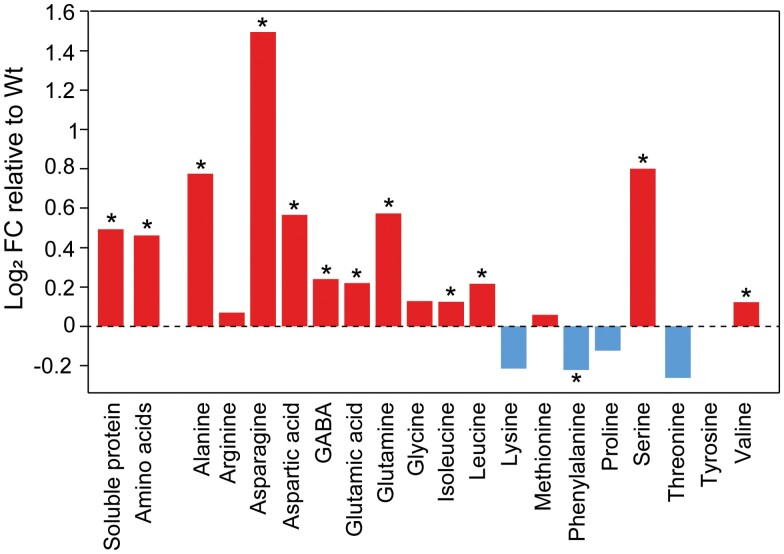
Log_2_ fold change (FC) of soluble protein and amino acid contents in o/exTrxm plants compared with the Wt. Leaf samples were taken after 4 h illumination. Significant differences between Wt and o/exTrxm are indicated by asterisks (*P<0.05, Student’s *t*-test).

By contrast, the amounts of soluble protein and total amino acids were higher in o/exTrxm leaves (~40%) compared with the Wt (Fig. 3, Supplementary Table S2). To further analyze the impact of Trx m overexpression on N metabolism, the leaf free amino acid profile was analyzed. The concentrations of 10 amino acids were significantly higher in the o/exTrxm genotype: alanine, asparagine, aspartic acid, γ-aminobutyric acid, Glu, Gln, isoleucine, leucine, serine (Ser), and valine. In contrast, only phenylalanine (Phe) showed a significantly lower concentration, by ~14%, in the transplastomic plants compared with the Wt (Fig. 3, Supplementary Table S2). Therefore, both the observed decrease in starch and sugars and the increase in soluble protein and amino acids suggest that the overexpression of Trx m in tobacco chloroplasts induces an imbalance between C and N.

### Metabolomic analysis supports a C and N imbalance in the o/exTrxm genotype

GC-TOF-MS-based metabolite profiling was performed to investigate the global effect of Trx m overexpression on plant metabolism. A total of 44 metabolites were annotated, including amino acids, sugars, organic acids and other metabolites (Supplementary Table S3). Only few metabolites displayed statistically significant differences between o/exTrxm and the Wt in the GC-MS analysis due to high variation of the data (Supplementary Table S3). Nevertheless, several metabolites exhibited the same general trend as that shown in Figs 2 and 3. Sugars displayed the same general trend to decrease in both analyses, and the concentrations of alanine and Ser were both significantly increased while Phe was significantly decreased, as in the HPLC analysis (Figs 2, 3, Supplementary Table S2, Supplementary Table S3). Metabolites not overlapping with those detected by HPLC are shown in Table 1 as relative levels to Wt. Among the sugars, a general downward trend was observed in the o/exTrxm metabolome, with fucose being significantly decreased and maltose and trehalose being below the level of detection. Some organic acids, most of them related to the phenylpropanoid pathway, were also significantly lower in the o/exTrxm genotype, including threonic acid, *cis*-3-caffeoylquinic acid, *trans*-3-caffeoylquinic acid, and quinic acid. Altogether, the metabolomic results corroborate that the overexpression of Trx m in chloroplasts alters C and N partitioning in tobacco plants, promoting N (rather than C) metabolites and also decreasing the contents of phenylpropanoid precursors.

**Table 1.** Changes in leaf metabolite levels after 4 h illumination in o/exTrxm plants relative to Wt

	Wt	Trx m
*Sugars*		
1,6-anhydro-beta-d-glucose	1±0.15	0.96±0.07
dl-rhamnose	1±0.13	0.90±0.06
**dl** **-fucose**	**1±0.18**	**0.53±0.04**
Gentiobiose	1±0.24	0.48±0.08
d-xylose	1±0.30	0.32±0.05
**d** **-maltose**	**1±0.03**	**n.d.**
**α-α′-** **d** **-trehalose**	**1±0.31**	**n.d.**
*Organic acids*		
*trans*-Caffeic acid	1±0.25	1.30±0.09
Nicotinic acid	1±0.09	1.18±0.20
Citric acid	1±0.21	1.13±0.40
Fumaric acid	1±0.18	1.12±0.18
*n*-Nonanoic acid	1±0.22	0.93±0.14
4-Aminobutyric acid	1±0.11	0.87±0.16
Pyruvic acid	1±0.15	0.86±0.25
Succinic acid	1±0.16	0.86±0.07
dl-malic acid	1±0.18	0.85±0.17
Phosphoric acid	1±0.32	0.60±0.19
2-Oxoglutaric acid	1±0.21	0.41±0.13
**Threonic acid**	**1±0.15**	**0.39±0.09**
***cis*-3-Caffeoylquinic acid**	**1±0.18**	**0.35±0.06**
***trans*-3-Caffeoylquinic acid**	**1±0.19**	**0.25±0.06**
dl-glyceric acid	1±0.41	0.15±0.03
**d-** **(–)-quinic acid**	**1±0.37**	**0.08±0.02**
*Others*		
Putrescine	1±0.13	1.60±0.24
Myo-inositol	1±0.23	0.55±0.11
Tyramine	1±0.31	0.42±0.06

Metabolite profiling was performed using GC-TOF-MS analysis. Results are means ±SE (*n*=4). Values that are significantly different from the Wt according to Student’s *t*-test are indicated in bold (*P*< 0.05). n.d., Not detected.

### Activity of C and N metabolism enzymes

**Fig. 4.**
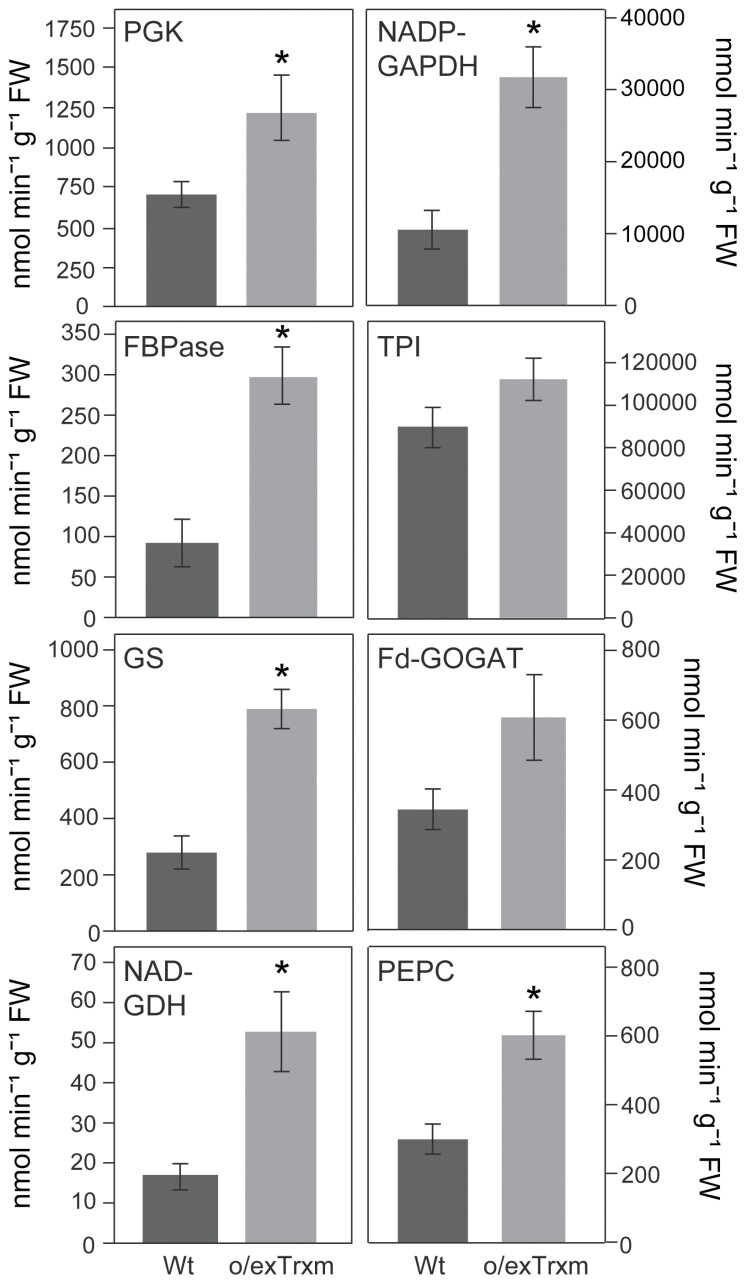
C and N metabolism enzyme activities in o/exTrxm tobacco leaves. Samples were harvested after 4 h illumination from the youngest fully expanded leaves of plants grown in a phytotron under a 16 h light photoperiod and assayed for phosphoglycerokinase (PGK), NADP-dependent glyceraldehyde-3-phosphate dehydrogenase (NADP-GAPDH), fructose-1,6-bisphosphatase (FBPase), triose-phosphate isomerase (TPI), glutamine synthetase (GS), ferredoxin-dependent glutamate synthase (Fd-GOGAT), NAD-dependent glutamate dehydrogenase (NAD-GDH), and phospho*enol*pyruvate carboxylase (PEPC). Results are the mean ±SE of five individual plants. Statistical significance compared with Wt plants is indicated by asterisks (*P<0.05, Student’s *t*-test).

We further analyzed the activity of several enzymes involved in primary C and N metabolism in Wt and o/exTrxm leaves harvested after a 4 h light period. Consistent with the proposal that Trx m plays an important role in the activation of CBC enzymes *in vivo* (Okegawa and Motohashi, 2015), a significant increase in PGK, NADP-GAPDH, and stromal FBPase activities was observed in the o/exTrxm genotype compared with the Wt, whereas the activity of TPI was not significantly different between the genotypes (Fig. 4). When the activity of N cycle-related enzymes was analyzed, the o/exTrxm genotype also showed a general increase in the activity of the GS-GOGAT cycle enzymes. GS and Fd-GOGAT activities were higher in o/exTrxm leaves, although this was statistically significant only for GS (Fig. 4). NAD-GDH and PEPC activities were also measured because of their central position at the interface between C and N metabolism. The o/exTrxm genotype displayed higher extractable activities for both of these enzymes (Fig. 4). It is also worth mentioning that all these enzyme activities were still increased when expressed on a protein basis (Supplementary Fig. S1). With the exception of FBPase activity, the observed increase in enzyme activity also seemed to be specific to Trx m because tobacco plants overexpressing its counterpart, Trx f (Sanz-Barrio *et al.*, 2013), did not show the same effect (Supplementary Table S4).

To assess whether the significant and specific increase observed in the enzyme activities of Trx m-overexpressing plants resulted from a transcriptional or post-transcriptional regulation, the levels of mRNA transcripts of genes encoding PGK, NADP-GAPDH, and GS2 were analyzed using RT–qPCR. The transcript levels of all these genes were unchanged in the o/exTrxm genotype compared with the Wt (Supplementary Fig. S2), revealing that their increased activity likely resulted from a post-transcriptional regulation specifically promoted by the overexpression of Trx m in chloroplasts.

### Impact of Trx m overexpression on oligomerization, activity, and stability of GS2

**Fig. 5.**
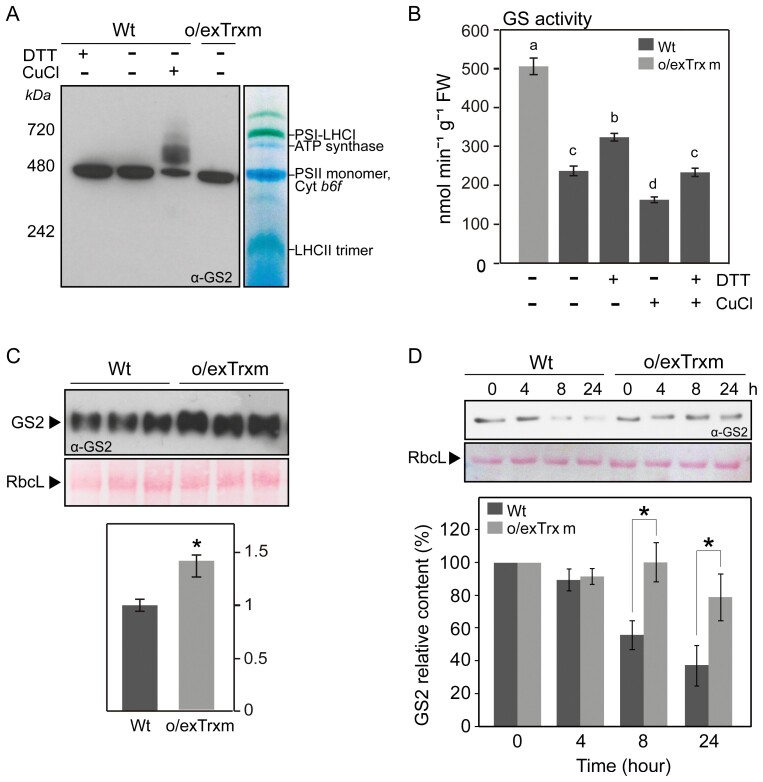
Determination of GS2 oligomerization, activity, and stability under oxidative conditions. (A) GS2 protein complexes. Proteins from Wt and o/exTrxm chloroplasts were extracted under non-reducing conditions, separated by BN-PAGE, and analyzed by Western blot with anti-GS2 antibody. Photosynthetic thylakoid complexes are shown on the right and the molecular weight marker on the left. (B) Redox-sensitive GS activity. Protein extracts from Wt plants were incubated with or without 5 mM DTT or 100 µM CuCl_2_ for 30 min on ice, and the activity was then measured. Recovery of GS activity by reduction was performed by incubating with 5 mM DTT on ice after sample oxidation treatment (100 µM CuCl_2_). The activity of protein extracts from o/exTrxm plants was also measured. Results are expressed as means ±SE (*n*=3). (C) Quantification of GS2 in the o/exTrxm genotype. Total protein extracts (2.5 µg) were separated by SDS-PAGE and analyzed by western blot. Three different plants per line are shown in the blot. Immunoblots were analyzed with Gene Tools Analyzer software (SynGene). Data are given as means ±SE with the Wt relativized to 1. Statistical significance relative to the Wt is indicated by an asterisk (*P<0.05, Student’s *t*-test). (D) Degradation of GS2 protein in Wt and o/exTrxm plants. Leaves were incubated at 30 °C for up to 24 h in the presence of FeCl_3_, and the content of GS2 protein was analyzed by immunodetection. Similar results were obtained in eight independent biological replicates, and a representative blot is shown (upper panel). Immunoblots were analyzed with Gene Tools Analyzer software. Protein levels in plants treated for 4, 8, and 24 h are shown relative to the levels in plants treated for 0 h. Data are given as means ±SE. Statistical significance relative to Wt plants is indicated by asterisks (*P<0.05, Student’s *t*-test).

We further investigated the oligomerization, activity, and stability of GS2 in o/exTrxm and Wt plants (Fig. 5). Since GS2 polypeptides must be assembled into decameric complexes to be active (Unno *et al.*, 2006), we analyzed whether the overexpression of Trx m in the chloroplast might affect the formation of GS2 decamers (~420 kDa). BN-PAGE analyses, followed by immunoblotting, were performed on protein extracts from o/exTrxm and Wt chloroplasts. Our results showed that the oligomeric GS2 enzyme migrated with a size compatible with a decameric structure in both Wt and o/exTrxm plants (Fig. 5A). Similarly, GS2 decamers were not affected by DTT treatment, while significant structural changes (aggregation) were observed when chloroplast proteins were oxidized with CuCl_2_ (Fig. 5A). As indicated before (Fig. 4), GS activity was higher in o/exTrxm than in Wt plants, but we also noticed that the GS activity in the Wt genotype was increased by DTT treatment and decreased when protein extracts were incubated with the oxidant CuCl_2_ (Fig. 5B). In addition, GS activity was restored when protein extracts were incubated with 5 mM DTT after an oxidation treatment (100 µM CuCl_2_ for 30 min) (Fig. 5B).

The amount of GS2 protein was clearly increased in o/exTrxm plants compared with the Wt (by 40%; Fig. 5C). As shown before (Supplementary Fig. S2), GS2 transcripts were unaltered in the o/exTrxm genotype, suggesting that the overexpression of Trx m might have an influence on maintaining GS2 stability. The stability of GS2 was evaluated in the presence of the oxidizing agent FeCl_3_ (Fig. 5D). In the Wt genotype, the intensity of the immunoreactive GS2 band decreased by 45% and 62% after 8 h and 24 h of incubation, respectively, relative to 0 h, whereas in the o/exTrxm genotype, a decrease of ~20% was visible only after 24 h. These results indicate that the overexpression of Trx m led to increased activity and stability of GS2.

### Integrating C and N metabolisms with photorespiration and respiration

**Fig. 6.**
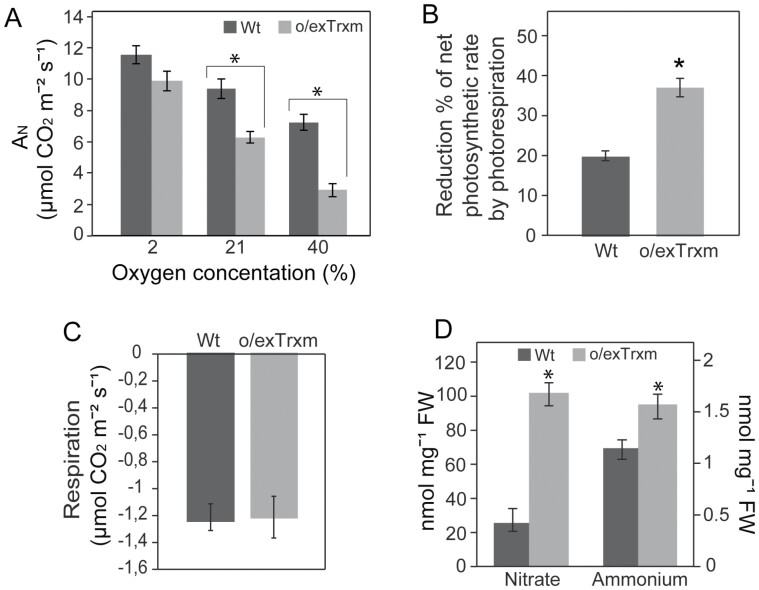
Net photosynthesis, photorespiration, respiration, and N status in o/exTrxm tobacco plants. (A) Gas exchange measurements at different oxygen concentrations (2%, 21%, and 40% O_2_). A_N_, net CO_2_ uptake rate. (B) Percentage of reduction in the net photosynthetic rate by photorespiration. The asterisk indicates a statistically significant difference (*P<0.05, Student’s *t*-test). (C) Dark respiration rates in Wt and o/exTrxm tobacco plants grown under a 16 h light photoperiod. (D) Nitrate and ammonium concentrations in Wt and o/exTrxm plants harvested after 4 h illumination. Results are the mean ±SE of six individual plants. Asterisks indicate statistically significant differences (*P<0.05, Student’s *t*-test).

We further analyzed the photorespiration in the o/exTrxm genotype, given its influence over the interaction between C and N metabolisms. Gas exchange analyses showed that net photosynthesis under ambient conditions (A_N_, determined at 21% O_2_) decreased significantly (by ~30%) in o/exTrxm plants relative to the Wt, whereas no significant differences in CO_2_ fixation rates were observed between the genotypes when analyzed at ~2% O_2_ (A_N2%_; Fig. 6A). Moreover, the reduction of net photosynthesis in the o/exTrxm genotype compared with the Wt (by ~60%) was greatly intensified at the higher O_2_ concentration (A_N40%_; Fig. 6A), indicating a higher flux through photorespiration. Therefore, the reduction of net photosynthetic rate by photorespiration in the o/exTrxm genotype (36±2%) was twice as high as that observed in the Wt (19±2%; Fig. 6B). These results indicate that photorespiration most likely contributes to the decrease in net photosynthesis observed in the o/exTrxm genotype. However, no significant differences in dark respiration (R_d_) were detected between the genotypes (Fig. 6C). Because photorespiration is intimately associated with N assimilation, the concentrations of nitrate and ammonium were also analyzed in these plants. The concentrations of ammonium and, more importantly, nitrate were significantly higher in the o/exTrxm genotype than in the Wt (Fig. 6D). These results collectively indicate that the overexpression of Trx m in chloroplasts also alters the so-called photorespiratory N cycle.

### Influence of Trx m overexpression in plant development under N-limiting conditions

**Fig. 7.**
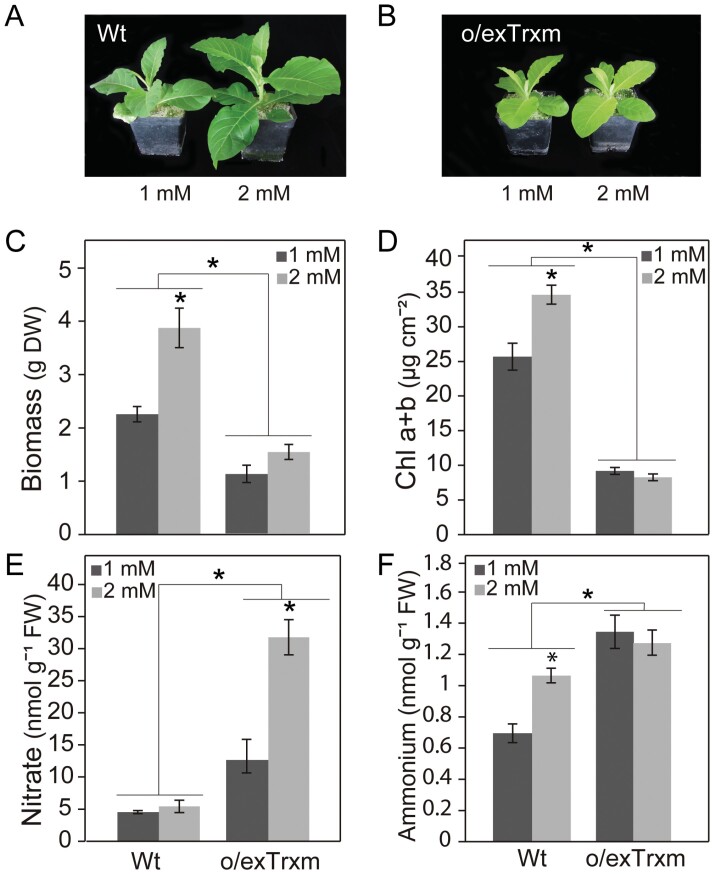
Response to nitrate supply. Plants were grown under a 16 h light photoperiod and watered daily with 1 mM or 2 mM KNO_3_. After 30 days, plants were photographed (A, B), harvested after 4 h illumination, and analyzed for (C) whole plant biomass, (D) total chlorophyll, (E) nitrate concentration, and (F) ammonium concentration. Results are the mean ±SE of four individual plants. Statistical significance between treatments is indicated by asterisks (*P<0.05, Student’s *t*-test).

Given that overexpression of Trx m in chloroplasts affected the N status of tobacco plants, we next analyzed the influence of nitrate supply on plant growth. Wt and o/exTrxm plants were grown in perlite and irrigated with nutrient solution containing low concentrations of N (1 or 2 mM KNO_3_). In all assayed conditions, the o/exTrxm genotype exhibited slower growth and lower chlorophyll content compared with the Wt (Fig. 7A–D), as previously reported (Rey *et al.*, 2013; Sanz-Barrio *et al.*, 2013). However, we observed a significant decrease in the biomass of Wt plants grown at 1 mM nitrate compared with 2 mM nitrate, which did not occur in the o/exTrxm plants (Fig. 7A–C). The same phenomenon was observed when chlorophyll content was measured (Fig. 7D), indicating that low N supply has a greater impact on plant performance in Wt than in o/exTrxm plants. It should be noted that, under N-limiting conditions, o/exTrxm leaves also accumulated more nitrate and ammonium than Wt leaves (Fig. 7E, F). In fact, Wt leaf nitrate concentrations were much lower than in plants irrigated with full nutrient solution (Fig. 6D, Fig. 7E), whereas they were still high in the o/exTrxm genotype grown under these conditions. Conversely, leaf ammonium concentrations appeared to be less sensitive to N deficiency because only Wt plants grown at 1 mM nitrate showed a significant decrease in ammonium concentration (Fig. 6D, Fig. 7F). Together, these results indicate that the o/exTrxm phenotype was relatively unaffected by the N-limiting conditions.

## Discussion

C and N assimilation in leaves requires the partitioning of reductant and photosynthates to sustain the demands of amino acid and carbohydrate biosynthesis. These processes are regulated at different levels according to the prevailing environmental conditions. The ability of plants to coordinate C and N metabolism consists of both a complex sensory system and an intricate associated signaling network (Nunes-Nesi *et al.*, 2010). Recent advances are providing more evidence that modulation of the redox status of the plant might be an important signaling mechanism for the coordination of C and N assimilation (Chaput *et al.*, 2020), with chloroplast Trxs serving as crucial components of the redox network (Schürmann and Buchanan, 2008). In the current study, we characterized the C and N metabolism of tobacco plants overexpressing Trx m from the chloroplast genome, and we found that Trx m could be related to the regulation of N metabolism.

### C and N imbalance is associated with Trx m overexpression in tobacco chloroplasts

Tobacco plants overexpressing Trx m exhibited altered C and N partitioning, promoting the accumulation of N-related metabolites at the cost of C-related ones (Figs 2, 3, Table 1). This effect seems to be specific to the m-type Trx because it does not occur when Trx f is overexpressed at the same levels (Sanz-Barrio *et al.*, 2013; Aranjuelo *et al.*, 2015). Several enzymes of the starch degradation pathway have been reported as being redox activated by Trxs (Skryhan *et al.*, 2018). However, this is an unlikely explanation for the low starch content reported in this genotype, because the major starch breakdown products (maltose and glucose; Zeeman *et al.*, 2010) did not accumulate in the o/exTrxm genotype (Fig. 2, Table 1). The lower photosynthetic rates of o/exTrxm (Fig. 6A) could, furthermore, have contributed to the observed reduction in carbohydrates. To identify candidates that might explain the down-regulation of photosynthesis, the activities of some CBC enzymes were determined (Fig. 4). Curiously, these determinations revealed that the activities of PGK and NADP-GAPDH were higher in o/exTrxm than in Wt and o/exTrxf plants (Fig. 4, Supplementary Table S4). However, the higher activities of PGK and NADP-GAPDH were not in line with the lower photosynthetic rates. These results suggest that while Trx m seems to redox activate CBC enzymes, as previously reported (Okegawa and Motohashi, 2015), the limitation in net photosynthesis in the o/exTrxm genotype could rather be associated with an impairment of the photosynthetic machinery (Ancín *et al.*, 2019) that produces the reducing power and energy necessary for CO_2_ fixation.

While C metabolism was impaired, our study showed that overexpression of Trx m favored N metabolism. Indeed, in addition to amino acids, the soluble protein concentration was increased in the o/exTrxm genotype relative to the Wt (Fig. 3, Supplementary Table S2). Amino acid concentrations basically depend on their relative rates of production and consumption and/or transport (Nunes-Nesi *et al.*, 2010). Several enzymes involved in amino acid biosynthesis have been postulated as potential Trx targets in the spinach chloroplast (Balmer *et al.*, 2003) or in *Chlamydomonas reinhardtii* (Lemaire *et al.*, 2004), and some of these enzymes are involved in the synthesis of the amino acids that were found to be increased in concentration in the o/exTrxm genotype (Fig. 3, Supplementary Table S2). Therefore, given that the overexpressed Trx m is in the reduced form in this genotype (Fig. 1C), a direct Trx m-dependent regulation of some of these chloroplastic enzymes cannot be ruled out. Moreover, the general increase in amino acids and, in particular, the higher Glu and Gln pools demonstrated in the o/exTrxm genotype (Fig. 3, Supplementary Table S2) may be indicative of an up-regulation of the GS-GOGAT cycle via post-translational reductive activation, as shown by Kolbe *et al.* (2006) in Arabidopsis leaves treated with DTT. In fact, our results revealed a significant increase in GS activity in the o/exTrxm genotype relative to the Wt (Figs 4, 5B). Hence, the increased amino acid concentrations observed in the present study could be caused by a reductive activation of the GS-GOGAT cycle *in vivo*, exerted by the overexpressed Trx m. The mitochondrial GDH, which operates at the interface of C and N metabolism and participates in the balancing of the cellular levels of three major components (ammonium, 2-oxoglutarate, and Glu), also exhibited higher activity in the o/exTrxm genotype (Fig. 4). Assuming that in leaves, GDH, which is coupled to respiration, mostly operates in the deaminating direction (Fontaine *et al.*, 2012), one hypothesis is that the flux through the futile cycle involving GS, Fd-GOGAT, and GDH could be increased in the o/exTrxm genotype. Such a cycle would be in line with sugar depletion (energy is mostly used by the reactions catalyzed by GS and Fd-GOGAT) and with the increased concentrations of nitrate and ammonia, as well as increased photorespiration. Indeed, more N would be cycled and less N would be used for growth, even if the amino acid and protein contents increased. Altogether, our results suggest that the overexpression of Trx m is involved in the control of the cellular C/N balance.

### Trx m overexpression leads to an increase in GS2 activity and stability

Our findings showed that the overexpression of Trx m in tobacco chloroplasts caused an increase in both the activity and content of GS2 (Fig. 4, Fig. 5B, C), while transcript levels were unaltered (Supplementary Fig. S2). The observed increase in GS2 protein content could mainly contribute to the increase in GS activity, but a post-translational regulation of GS2 in the o/exTrxm genotype, improving its stability or activity (or both), cannot be ruled out. Supporting this hypothesis, Choi *et al.* (1999) showed that the addition of reducing agents, especially DTT, increases the activity of recombinant GS2 from *Canavalia lineata*. These authors demonstrated that the redox sensitivity of GS2 is due to two conserved Cys residues found exclusively in the chloroplast isoform, although GS2 with mutations in one of these Cys residues still retained its redox activation. In agreement with these observations, the activity and stability of GS2 from *Lotus japonicus*, in which only one of the two conserved Cys residues is preserved, were considerably improved in the presence of thiol compounds (Betti *et al.*, 2006). It should be noted that GS2 from tobacco also conserves only one of these Cys residues (Supplementary Fig. S3), indicating that this unique conserved Cys should be sufficient for the redox activation of GS2, as occurs in *L. japonicus* plants. However, we have not been able to demonstrate a direct redox activation of GS2 in the o/exTrxm genotype.

The analysis of the quaternary structure revealed that the o/exTrxm genotype maintained the ability to form GS2 decamers (Fig. 5A), the protein complex known to be catalytically active in chloroplasts (Unno *et al.*, 2006; Kimata-Ariga and Hase, 2014). However, the complex formation, activity, and protein stability of GS2 seemed to be affected by oxidants (Fig. 5A, B, D). In fact, it was previously demonstrated that GS2 displays an extreme sensitivity to oxidants in plants, resulting in its aggregation and cleavage (Palatnik *et al.*, 1999; Ishida *et al.*, 2002; Castro-Rodríguez *et al*., 2015). In this line, it is possible that an indirect protection of GS2 against cellular oxidants exists in the o/exTrxm genotype. On the one hand, peroxiredoxins (Prxs) have been proposed as principal actors in the mechanism protecting GS from oxidative inactivation (Cheong *et al.*, 1999; Rahlfs and Becker, 2001; Jeon and Ishikawa, 2003; Liao *et al.*, 2017). In this way, Trx m may be activating this protection system through Prxs, given that multiple Prxs have been reported as Trx targets in chloroplasts (Montrichard *et al.*, 2009). In fact, we saw that 2-Cys Prx was more efficiently reduced in o/exTrxm than in Wt plants in both dark and light conditions (Supplementary Fig. S4), accompanied by a reduced H_2_O_2_ content (Ancín *et al.*, 2019). On the other hand, Trxs provide reducing power to methionine sulfoxide reductases (MSRs), enzymes that repair oxidized proteins (Vieira Dos Santos *et al.*, 2007), which could likewise function to up-regulate the biological activity of GS protein (Levine *et al.*, 2000). Interestingly, we have previously reported a 2-fold higher MSR enzymatic capacity in o/exTrxm plants compared with the Wt (Rey *et al.*, 2013). These findings suggest that an antioxidant protective mechanism might be affecting the activity and stability of GS2 in o/exTrxm plants.

### Overexpression of Trx m in chloroplasts favors the photorespiratory N cycle rather than nitrate assimilation

We have recently demonstrated that a reduction in the electron transport rate in the o/exTrxm genotype may be related to the reduced photosynthetic capacity in this genotype (Ancín *et al.*, 2019). However, our current results support that changes in photorespiration also contributed to the decreased net photosynthesis. In fact, the net photosynthesis in the o/exTrxm genotype was Wt-like at 2% O_2_ but greatly decreased at higher O_2_ concentrations (21% and 40%; Fig. 6A). Accordingly, we observed a much higher percentage of A_N_ reduction by photorespiration in the o/exTrxm genotype than in the Wt (Fig. 6B). Photorespiration is known to interact with several primary metabolic pathways (Hodges *et al.*, 2016), including the GS-GOGAT cycle (Coschigano *et al.*, 1998; Keys, 2006). The conversion from glycine (Gly) to Ser in the photorespiratory cycle results in the production of ammonia at a rate far exceeding that of primary N assimilation, by up to 10-fold (Keys *et al.*, 1978). Meanwhile, GS2 controls the reassimilation of the released ammonia in chloroplasts (Nunes-Nesi *et al.*, 2010), a process known as the photorespiratory N cycle. Interestingly, the pool of Ser was higher in the o/exTrxm genotype than in the Wt, while the pool of Gly remained unaltered (Fig. 3Supplementary Table S2), indicating a higher rate of Gly to Ser conversion that correlates with a significant increase in the ammonium concentration in o/exTrxm leaves (Fig. 6D). Although three different pathways of Ser biosynthesis have been described in plants (the glycolate, phosphorylated, and glycerate pathways), the glycolate pathway, which is associated with photorespiration, is considered to be the most important source of Ser, at least in photosynthetic tissues (Ros *et al.*, 2014; Igamberdiev and Kleczkowski, 2018). Therefore, the higher amount of Ser observed in o/exTrxm plants was very likely coming from an alteration of the glycolate pathway. Given the low glycerate levels observed in o/exTrxm plants (Table 1), the glycerate pathway could hardly contribute to increase the Ser pool. The phosphorylated pathway is more relevant at night and under conditions in which the photorespiratory pathway does not function (Benstein *et al.*, 2013; Toujani *et al.*, 2013). However, a putative activation of this pathway in o/exTrxm plants cannot be ruled out given that the enzyme that catalyzes the first reaction of this route, 3-PGA dehydrogenase, was previously identified as a Trx target by proteomic approaches (Balmer *et al.*, 2003; Alkhalfioui *et al.*, 2007). Therefore, the enhanced photorespiratory capacity in the transplastomic genotype may be linked to the activation of the GS-GOGAT cycle. The reassimilation of ammonia into chloroplasts is accurately regulated by the transport of dicarboxylates across the chloroplast envelope via the 2-oxoglutarate/malate transporter (OMT or DiT1) and the general dicarboxylate transporter (DCT or DiT2) (Taniguchi *et al.*, 2002; Renné *et al.*, 2003). DiT1 and DiT2 constitute a double translocator system that functions to coordinate the photorespiration and GS-GOGAT pathways (Weber and Flügge, 2002; Kinoshita *et al.*, 2011). We demonstrated by qPCR that the o/exTrxm genotype showed an increase in the expression of *DiT2* relative to the Wt (Supplementary Fig. S5), thus reinforcing our proposal of an up-regulated photorespiratory N cycle in these plants.

According to Nunes-Nesi *et al.* (2010), the occurrence of photorespiratory N recycling under photorespiratory conditions may result in a lower net primary rate of inorganic N assimilation in the leaf, pointing to a stimulated use of the photorespired ammonium. In agreement with this hypothesis, the o/exTrxm genotype accumulated extremely large amounts of nitrate in leaves (Fig. 6D), a phenotype that resembles that of nitrate reductase (NR) mutant plants (Scheible *et al.*, 1997). In addition, we showed that the o/exTrxm genotype was less sensitive than the Wt to nitrate deprivation (Fig. 7). This behavior could be due to an inhibition of the NR enzyme, causing a deficiency in nitrate reduction. Because NR is located in the cytosol, the overexpression of Trx m might exert indirect control over its activity. It is well known that a decrease in the availability of reductant in the cytosol may limit NR activity (Kaiser et al., 2000, 2002; Hashida *et al.*, 2018). Interestingly, a decrease in the NADPH/NADP^+^ ratio and the size of the NADP pool was observed in the o/exTrxm genotype (Supplementary Fig. S6), indicating a reduced flux of reducing power to the cytosol, which may explain the large accumulation of nitrate that occurred in o/exTrxm leaves. However, other factors occurring in the o/exTrxm genotype may also account for nitrate accumulation via a down-regulation of NR activity. Among them, sugar depletion (Kaiser and Huber, 2001) and decreases in net photosynthesis (Quick *et al.*, 1991) have been reported to result in a massive reduction in NR protein levels. Our results collectively suggest that the overexpression of Trx m in chloroplasts results in the increased reassimilation of photorespired ammonium while primary nitrate assimilation decreases.

### Putative role of Trx m in phenylpropanoid metabolism

Central metabolism is an important source of precursors for the synthesis of secondary metabolites. There are several lines of evidence that the range and levels of secondary metabolites are regulated in response to the C and N status in tobacco (Stitt *et al.*, 2002; Fritz *et al.*, 2006). Phenylpropanoids represent a diverse group of secondary metabolites, with roles in plant structure, defense, and signaling (Dixon and Paiva, 1995). They are synthesized via the shikimate pathway in the plastid, with aromatic amino acids as precursors. In the o/exTrxm genotype there was a dramatic decrease in the concentrations of two shikimate pathway-related compounds, namely quinic and caffeoylquinic acids (Table 1), the latter being one of the predominant soluble phenylpropanoids in the Solanaceae (Vogt, 2010). This result is in accordance with Fritz *et al.* (2006), who demonstrated a stimulation of phenylpropanoid metabolism in response to low nitrate. Hence, the higher accumulation of nitrate (Fig. 6D) in the o/exTrxm genotype could account for the decrease in quinic and caffeoylquinic acids. Moreover, phenylpropanoids are synthesized from Phe, and we also found a specific decrease in the concentration of this amino acid in the o/exTrxm genotype (Fig. 3, Supplementary Table S2). Alternatively, the decreased phenylpropanoid content in the o/exTrxm genotype could be related to a decrease in the activity of the phospho*enol*pyruvate (PEP) translocator (Henkes *et al.*, 2001). Interestingly, an increase in the activity of PEPC, which catalyzes the conversion of PEP and HCO_3_^–^ to oxaloacetate (OAA) and inorganic phosphate, occurred in these plants (Fig. 4). OAA is the precursor of most of the amino acids that are increased in the o/exTrxm genotype. Hence, our results suggest that in the o/exTrxm genotype PEP preferentially serves as PEPC substrate instead of being exported through the PEP translocator to the chloroplast, thus negatively affecting the phenylpropanoid content. In agreement with this, Arabidopsis PEPC mutants showed a large reduction in the flux from PEP to OAA and, thereby, suppression of the GS-GOGAT cycle and subsequent ammonium assimilation (Shi *et al.*, 2015), a phenotype completely opposite to that of the o/exTrxm genotype.

Here, we show that when Trx m is overexpressed in tobacco chloroplasts the observed decreases in the rate of photosynthesis and carbohydrate concentrations is not accompanied by an inhibition of N metabolites; on the contrary, there is an increase in amino acids (with the exception of Phe) and protein content. These results differ from those obtained in plants with a decreased rate of photosynthesis, where the levels of carbohydrates decrease along with those of the N-derived compounds (Henkes *et al.*, 2001; Matt *et al.*, 2002). Our results, combined with those of published studies, point towards a specific influence of Trx m inside the chloroplast, regulating the so-called photorespiratory N cycle and the activity and stability of GS2, thereby unbalancing C and N partitioning in plants.

## Supplementary data

The following supplementary data are available at *JXB* online.

Fig. S1. Enzyme activities in o/exTrxm tobacco plants expressed on a protein basis.

Fig. S2. RT–qPCR analysis of *gln2*, *pgk*, and *gapB* expression.

Fig. S3. Alignment of amino acid sequences of GS2.

Fig. S4. Redox status of 2-Cys Prx in o/exTrxm plants.

Fig. S5. RT–qPCR analysis of *DiT1* and *DiT2* expression.

Fig. S6. Effect of Trx m overexpression on the pyridine nucleotide content.

Table S1. Parameters used for peak annotation.

Table S2. Soluble protein and amino acid contents in Wt and o/exTrxm plants.

Table S3. Changes in the levels of the 44 annotated metabolites.

Table S4. Enzyme activities in plants overexpressing Trx f from the chloroplast genome.

erab193_suppl_Supplementary_Figures_S1-S6_Tables_S2-S4

erab193_suppl_Supplementary_Table_S1

## Data Availability

All data supporting the findings of this study are available within the paper and within its supplementary data published online.
