# Supplementary material for: Overexpression of thioredoxin m in chloroplasts alters carbon and nitrogen partitioning in tobacco
Source: J Exp Bot. 2021 May 8;72(13):4949–64. doi: 10.1093/jxb/erab193 (PMC8219043; doi:10.1093/jxb/erab193)
Supplement: erab193_suppl_Supplementary_Figures_S1-S6_Tables_S2-S4 [file erab193_suppl_supplementary_figures_s1-s6_tables_s2-s4.pdf]

Thioredoxin m overexpression in chloroplasts alters carbon and nitrogen partitioning in tobacco plants

María Ancín[1], Luis Larraya[1], Igor Florez-Sarasa[2,3], Camille Bénard[4], Alicia Fernández-San Millán[1], Jon Veramendi[1], Yves Gibon[4], Alisdair R. Fernie[2], Iker Aranjuelo[5] and Inmaculada Farran[1*]

[1]Institute for Multidisciplinary Applied Biology, Dpto. Agronomía, Biotecnología y Alimentación, Universidad Pública de Navarra, Campus Arrosadia, 31006 Pamplona, Spain; [2]Max-Planck-Institut für Molekulare Pflanzenphysiologie, Am Mühlenberg 1, 14476 Potsdam-Golm, Germany; [3]Centre for Research in Agricultural Genomics (CRAG) CSIC-IRTA-UAB-UB, Campus UAB Bellaterra, Barcelona, Spain; [4]UMR1332 Biologie du Fruit et Pathologie and Plateforme Métabolome Bordeaux, INRA, Bordeaux University, Villenave d'Ornon, France; [5]Instituto de Agrobiotecnología, CSIC-Gobierno de Navarra, Avda. Pamplona 123, 31192 Mutilva, Spain.

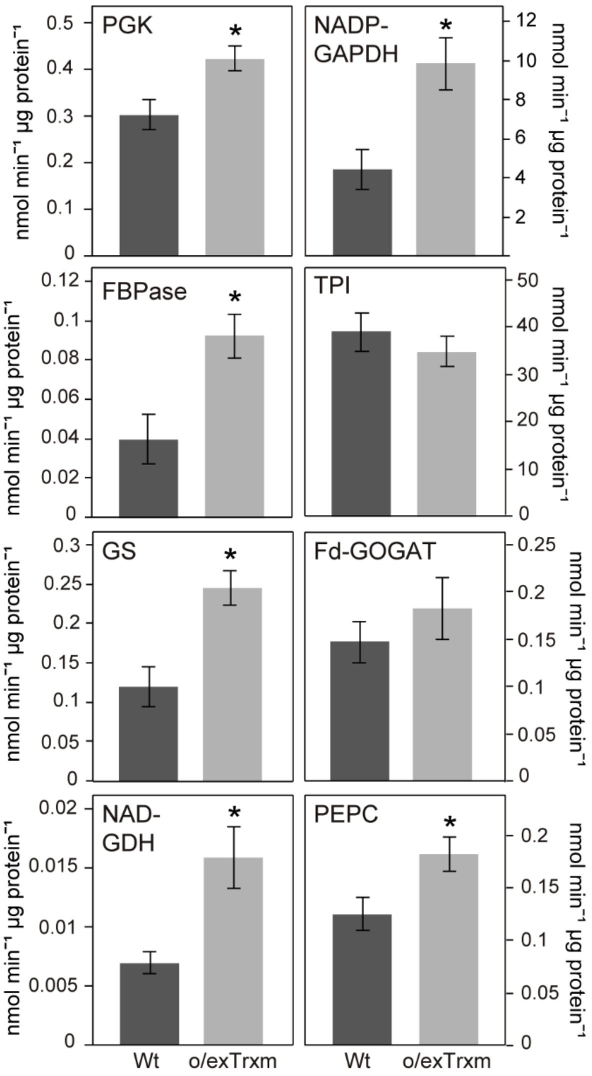

Figure S1. Enzyme activities in o/exTrxm tobacco plants expressed on a protein basis. Samples were harvested after 4 h illumination from the youngest fully expanded leaves of plants grown in a phytotron under a 16 h light photoperiod and assayed for phosphoglycerokinase (PGK), NADP-dependent glyceraldehyde-3-phosphate dehydrogenase (NADP-GAPDH), fructose-1,6-bisphosphatase (FBPase), triose-phosphate isomerase (TPI), glutamine synthetase (GS), ferredoxin-dependent glutamate synthase (Fd-GOGAT), NAD-dependent glutamate dehydrogenase (NAD-GDH) and phospho*enol*pyruvate carboxylase (PEPC). Results are the mean ± SE of five individual plants. Statistical significance compared to Wt plants is indicated by asterisks (*$P < 0.05$, Student's *t*-test).

A

Primers [a]	Forward (5'-3')	Reverse (5'-3')
gln2 (XM_016584731.1)	GCTTCAAACATGGACCCCTA	TTTGAGCAGCAAGAGCTTCA
pgk (Z48977.1)	CAAGGCGAAAGGAGTCAGTC	GATAGCAGATGCAGGCACAA
gapB (M14418.1)	TGTGTGACATTTTCCAACAAAG	AACAGGGGTTGATCTTGTGG
16S rRNA (Z00044.2)	CTTTTTAAGTCCGCCGTCAAA	TCTTTCCGATCTCTACGCATTTC
actin (XM_016628756.1)	CAGCAACTGGGATGATATGG	GGCGCTTCAGTAAGGAGGAC

[a]GenBank accession numbers (NCBI) are indicated in parenthesis

B

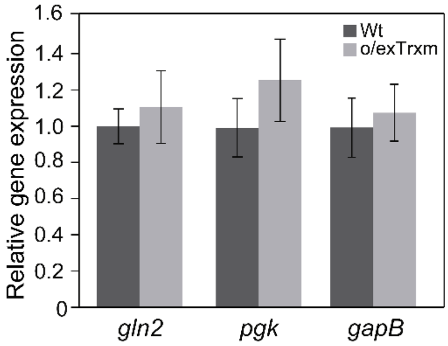

Figure S2. RT-qPCR analysis of *gln2*, *pgk* and *gapB* expression in tobacco Wt and o/exTrxm plants. Quantitative PCR analysis was carried out using gene-specific primers listed in (A). Relative expression levels were normalized to *16s rRNA* and *actin* expression. Graphical representation (B) shows the fold change as the mean of the different biological repeats (n=3). Error bars represent SE. No significant differences in expression levels of these genes between lines were found.

```
A.thaliana       MAQILAASPTCQMRVPKHSSV-IASSSKLWSSVVLKQKKQSN-NKVRGFRVLALQSDNST
C.lineata        MAQILAPSTQWQMRITKTSPNASPVTSNMWSSLLMKQNKKAT--SSAKFRVLAIKSENGT
N.tabacum        MAQILAPSAQWQMRMTKSSTDANPLTSKMWSSVVLKQNKRLAVKSSAKFRVFALQSDSGT
L.japonicus      MAQILAPSTQWQTRITKTSPNASPITSNMWSSLLWKQNKKVA--RASKFRVLAIKSDGST

A.thaliana       VNRVETLLNLDTKPYSDRIIAEYIWIGGSGIDLRSKSRTIEKPVEDPSELPKWNYDGSST
C.lineata        INRLENLLDLDITPYTDKIIAEYIWVGGTGIDVRSKSRTIARPVEHPSELPKWNYDGSST
N.tabacum        VNRVEQLLNLDVTPYTDKIIAEYIWIGGSGIDMRSKSRTISKPVKHASELPKWNYDGSST
L.japonicus      INRLEGLLNLDVTPYTDKFIAEYIWIGGTGVDVRSKSRTISKPVSHPSELPKWNYDGSST

A.thaliana       GQAPGEDSEVILYPQAIFRDPFRGGNNILVICDTWTPAGEPIPTNKRAKAAEIFSNKKVS
C.lineata        GQAPGDDSEVILYPQAIFKDPFRGGNNILVICDAYTPAGEPIPTNKRHRAAEIFSNPKVQ
N.tabacum        GQAPGEDSEVILYPQAIFKDPFRGGNNILVICDAYTPAGEPIPTNKRHKAAQIFSDSKVV
L.japonicus      GQAPGDDSEVILYPQAIFRDPFRGGNNILVICDAYTPQGEPIPTNKRHRAAEIFSNPKVQ

A.thaliana       GEVPWFGIEQEYTLLQQNVKWPLGWPVGAFPGPQGPYYCGVGADKIWGRDISDAHYKACL
C.lineata        AEVPWYGIEQEYTLLQTNVNWPLGWPVGGYPGPQGPYYCSAGADKSFGRDISDAHYKACL
N.tabacum        SEVPWFGIEQEYTLLQQNVKWPLGWPVGGYPGPQGPYYCGAGADKSFGRDISDAHYKACL
L.japonicus      AEIPWYGIEQEYTLLQTDVKWPLGWPVGGYPGPQGPYYCAAGADKSFGRDISDAHYKACL

A.thaliana       YAGINISGTNGEVMPGQWEFQVGPSVGIDAGDHVWCARYLLERITEQAGVVLTLDPKPIE
C.lineata        YAGINISGTNGEVMPGQWEFQVGPSVGIEAGDHIWAARYILERITEQAGVVLSLDPKPIQ
N.tabacum        YAGINISGTNGEVMPGQWEFQVGPSVGIEAGDHIWCARYILERITEQAGVVLSLDPKPIE
L.japonicus      YAGINISGTNGEVMPGQWEYQVGPSVGIEAGDHIWASRYLLERITEQAGVVLTLDPKPIE

                        *
A.thaliana       GDWNGAGCHTNYSTKSMREEGGFEVIKKAILNLSLRHKEHISAYGEGNERRLTGKHETAS
C.lineata        GDWNGAGCHTNYSTKSMREEGGFEVIKKAILNLSLRHSDHIRAYGEGNERRLTGKHETAD
N.tabacum        GDWNGAGCHTNYSTLSMREEGGFEVIKKAILNLSLRHKEHISAYGEGNERRLTGKHETAS
L.japonicus      GDWNGAGAHTNYSTKSMREEGGFEVIKKAILNLSLRHQDHIRAYGEGNERRLTGKHETAD

                             *
A.thaliana       IDQFSWGVANRGCSIRVGRDTEAKGKGYLEDRRPASNMDPYIVTSLLAETTLLWEPTLEA
C.lineata        INTFSWGVANRGCSIRVGRDTEKNGKGYLEDRRPASNMDPYVVTSLLAETTLLWEPTLEA
N.tabacum        IDKFSWGVANRGASIRVGRDTEKQGKGYLEDRRPASNMDPYVVTGLLAETTILWEPTLEA
L.japonicus      INTFSWGVANRGCSIRVGRDTEKEGKGYLEDRRPASNMDPYVVTALLAETTLLWEPTLEA

A.thaliana       EALAAQKLSLNV
C.lineata        EALAAQKIALKV
N.tabacum        EALAAQKLALNV
L.japonicus      EALAAQKIQLKV
```

Figure S3. Alignment of the amino acid sequence of GS2 from *Arabidopsis thaliana* (Q43127), *Canavalia lineata* (AAF17703), *Nicotiana tabacum* (XP_016440217) and *Lotus japonicus* (AAN84563), using the ClustalW software. The chloroplast-specific conserved cysteine residues (asterisks) are highlighted in a yellow box (Choi *et al*., 1999; Betti *et al*., 2006).

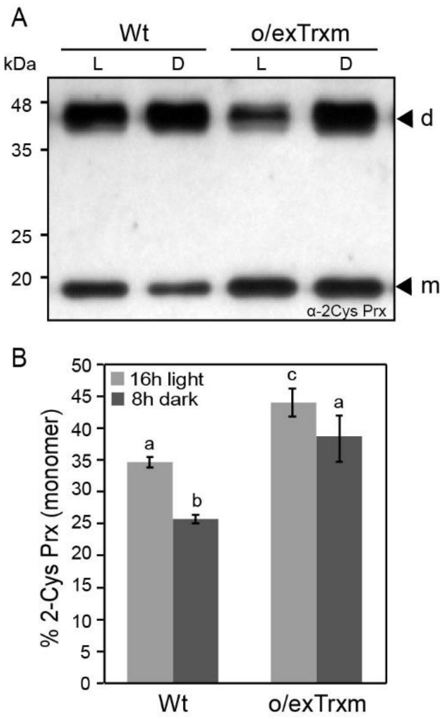

Figure S4. Redox status of 2-Cys Prx in o/exTrxm plants. Redox activation of 2-Cys Prx in leaves from o/exTrxm tobacco plants sampled at the end of the light (L) and dark (D) periods. A) A representative non-reducing western blot of 2-Cys Prx in leaves of Wt and o/exTrxm plants is shown. m: monomer; d: dimer. B) Quantification of 2-Cys Prx monomerization shown as the percentage of the 20 kDa monomer relative to the total amount of 2-Cys Prx. Results are the mean ± SE for four plants. Different letters above each bar indicate significant differences ($P < 0.05$, ANOVA).

A

Primers [a]	Forward (5'-3')	Reverse (5'-3')
DiT1 (XM_016606358.1)	GGGACTTGGTGCTTGTGTTT	GCAATTAACCATGGGATTGG
DiT2 (NM_001325346)	GATCTCCCGGATGTCTTCAA	GACCCAAGAACTTCCACCAA

[a]GenBank accession numbers (NCBI) are indicated in parenthesis

B

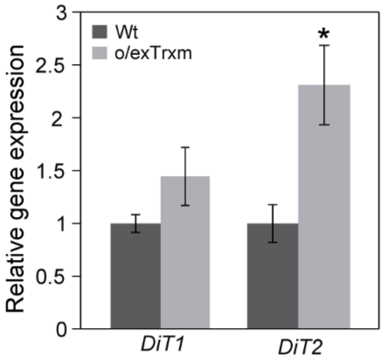

Figure S5. RT-qPCR analysis of *DiT1* and *DiT2* expression in tobacco Wt and o/exTrxm plants. Accumulation of mRNA was quantified by quantitative RT-PCR using the specific primers shown in (A). Relative expression levels were normalized to *16s rRNA* and *actin* expression. Graphical representation (B) shows the fold change as the mean of the different biological repeats (n=3). Error bars represent SE. Statistical significance compared with Wt plants is indicated by asterisks (*P < 0.05, Student's *t*-test).

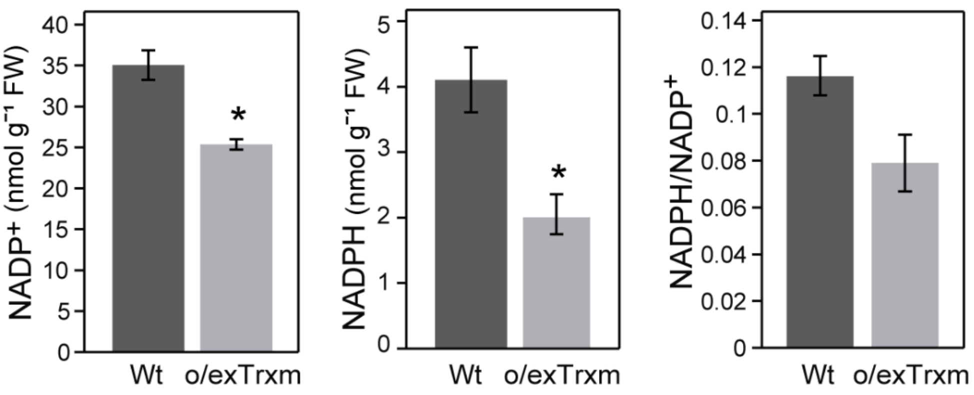

Figure S6. Effect of Trx m overexpression on the pyridine nucleotide content. Concentration of $NADP^+$ and NADPH (nmol g^{-1} FW) in Wt and o/exTrxm tobacco leaves after 4 h illumination measured as described in (Queval and Noctor, 2007; *Anal Biochem, 363:58-69*). Results are the mean ± SE of 4 individual plants. Statistical significance compared with Wt plants is indicated by asterisks (*P < 0.05, Student's *t*-test).

Table S2. Soluble protein and amino acid contents in Wt and o/exTrxm plants. Leaf samples were taken after 4 h illumination. Results are given in µg mg^{-1} FW for soluble protein and nmol g^{-1} FW for amino acids. Values are means ± SE (n=4-5). Values that are significantly different from Wt according to Student's *t*-test are indicated in bold (P value < 0.05).

	**Wt**	**o/exTrxm**	**P value**
Soluble Protein	6.4 ± 0.2	9.0 ± 0.4	**0.000**
Amino acids	5064.2 ± 167.2	6974.9 ± 296.0	**0.001**
Alanine	1265.3 ± 72.2	2166.0 ± 107.5	**0.000**
Arginine	98.9 ± 1.9	103.8 ± 1.7	0.111
Asparagine	110.0 ± 17.2	310.1 ± 41.7	**0.005**
Aspartic acid	399.7 ± 50.0	591.7 ± 19.9	**0.005**
GABA	234.4 ± 10.2	276.7 ± 11.9	**0.033**
Glutamic acid	845.5 ± 38.5	984.0 ± 38.1	**0.039**
Glutamine	196.7 ± 20.8	292.5 ± 17.0	**0.008**
Glycine	187.0 ± 8.4	204.4 ± 12.6	0.330
Isoleucine	96.3 ± 1.4	105.0 ± 2.0	**0.009**
Leucine	199.5 ± 6.7	231.7 ± 6.6	**0.010**
Lysine	55.8 ± 4.5	48.1 ± 2.5	0.187
Methionine	84.4 ± 1.1	87.9 ± 1.6	0.134
Phenylalanine	168.2 ± 3.4	144.2 ± 3.7	**0.002**
Proline	176.4 ± 13.7	162.6 ± 7.7	0.416
Serine	294.5 ± 26.0	513.0 ± 23.1	**0.000**
Threonine	571.7 ± 49.8	476.9 ± 88.8	0.430
Tyrosine	204.3 ± 6.0	204.3 ± 5.0	0.998
Valine	66.2 ± 1.6	72.1 ± 3.0	**0.022**

Table S3. Changes in the levels of the 44 annotated metabolites after 4 h illumination in o/exTrxm plants relative to Wt. Metabolite profiling was performed using GC-TOF-MS analysis. Results are means ± SE (n=4). Values that are significantly different from the Wt according to the Student's *t*-test are indicated in bold (P value < 0.05). n.d, not detected.

	**Wt**	**o/exTrxm**
Amino acids		
**DL-serine**	**1 ± 0.19**	**2.06 ± 0.27**
**DL-alanine**	**1 ± 0.09**	**1.76 ± 0.28**
DL-pyroglutamic acid	1 ± 0.25	1.45 ± 0.27
L-aspartic acid	1 ± 0.34	1.42 ± 0.30
DL-glutamine	1 ± 0.42	1.41 ± 0.19
DL-asparagine	1 ± 0.13	1.25 ± 0.16
DL-valine	1 ± 0.05	1.03 ± 0.11
DL-glutamic acid	1 ± 0.08	0.89 ± 0.11
L-isoleucine	1 ± 0.26	0.84 ± 0.30
DL-methionine	1 ± 0.11	0.69 ± 0.11
DL-threonine	1 ± 0.21	0.53 ± 0.28
**DL-phenylalanine**	**1 ± 0.07**	**0.49 ± 0.07**
Glycine	1 ± 0.32	0.49 ± 0.12
L-proline	1 ± 0.41	0.39 ± 0.14
Sugars		
1.6-anhydro-beta-D-glucose	1 ± 0.15	0.96 ± 0.07
DL-rhamnose	1 ± 0.13	0.90 ± 0.06
D-sucrose	1 ± 0.08	0.77 ± 0.12
**DL-fucose**	**1 ± 0.18**	**0.53 ± 0.04**
D-fructose	1 ± 0.31	0.48 ± 0.21
Gentiobiose	1 ± 0.24	0.48 ± 0.08
D-xylose	1 ± 0.30	0.32 ± 0.05
D-glucose	1 ± 0.53	0.17 ± 0.06
**D-maltose**	**1 ± 0.03**	**n.d**
**alpha-alpha'-D-trehalose**	**1 ± 0.31**	**n.d**
Organic acids		
trans-caffeic acid	1 ± 0.25	1.30 ± 0.09
Nicotinic acid	1 ± 0.09	1.18 ± 0.20
Citric acid	1 ± 0.21	1.13 ± 0.40
Fumaric acid	1 ± 0.18	1.12 ± 0.18
DL-malic acid	1 ± 0.06	1.01 ± 0.09
n-nonanoic acid	1 ± 0.22	0.93 ± 0.14
4-amino-butyric acid	1 ± 0.11	0.87 ± 0.16
Pyruvic acid	1 ± 0.15	0.86 ± 0.25
Succinic acid	1 ± 0.16	0.86 ± 0.07
DL-malic acid	1 ± 0.18	0.85 ± 0.17
Phosphoric acid	1 ± 0.32	0.60 ± 0.19
2-oxo-glutaric acid	1 ± 0.21	0.41 ± 0.13
**cis-3-caffeoylquinic acid**	**1 ± 0.18**	**0.35 ± 0.06**
**Threonic acid**	**1 ± 0.15**	**0.39 ± 0.09**
**trans-3-caffeoylquinic acid**	**1 ± 0.19**	**0.25 ± 0.06**
DL-glyceric acid	1 ± 0.41	0.15 ± 0.03
**D(-)-quinic acid**	**1 ± 0.37**	**0.08 ± 0.02**
Others		
Putrescine	1 ± 0.13	1.60 ± 0.24
myo-inositol	1 ± 0.23	0.55 ± 0.11
Tyramine	1 ± 0.31	0.42 ± 0.06

Fold over Wt
- 1.9-2
- 1.7-1.9
- 1.5-1.7
- 1.3-1.5
- 1.1-1.3
- 0.9-1.1
- 0.9-0.7
- 0.7-0.5
- 0.5-0.3
- 0.3-0.1
- 0.1-0

Table S4. Enzyme activities in plants overexpressing Trx f from the chloroplast genome (o/exTrxf). Samples were taken after 4 h illumination from leaves of plants grown in phytotron under 16h-light/8h-dark photoperiod and assayed for PGK, NADP-GAPDH, FBPase, TPI, GS, Fd-GOGAT, NAD-GDH and PEPC activities. Results, expressed as nmol min^{-1} g^{-1} FW, are the mean ± SE of five individual plants. Statistical significance compared with Wt plants is indicated by asterisks (*P < 0.05, Student's *t*-test).

	**PGK**	**NADP-GAPDH**	**FBPase**	**TPI**	**GS**	**Fd-GOGAT**	**NAD-GDH**	**PEPC**
**Wt**	706.9 ± 81.0	10472.7 ± 2679.2	92.0 ± 29.4	90539.5 ± 9493.0	279.2 ± 58.7	342.2 ± 58.2	16.9 ± 3.2	300.8 ± 43.9
**o/exTrxf**	545.9 ± 55.2*	9837.0 ± 2284.1	240.7 ± 62.0*	112228.6 ± 9964.7	256.7 ± 35.5	342.2 ± 18.5	20.6 ± 1.0	218.9 ± 16.2*
